# Supplementary figures and images for: Over-Expression of a 14-3-3 Protein From Foxtail Millet Improves Plant Tolerance to Salinity Stress in Arabidopsis thaliana
Source: Front Plant Sci. 2020 Apr 15;11:449. doi: 10.3389/fpls.2020.00449 (PMC7174642; doi:10.3389/fpls.2020.00449)

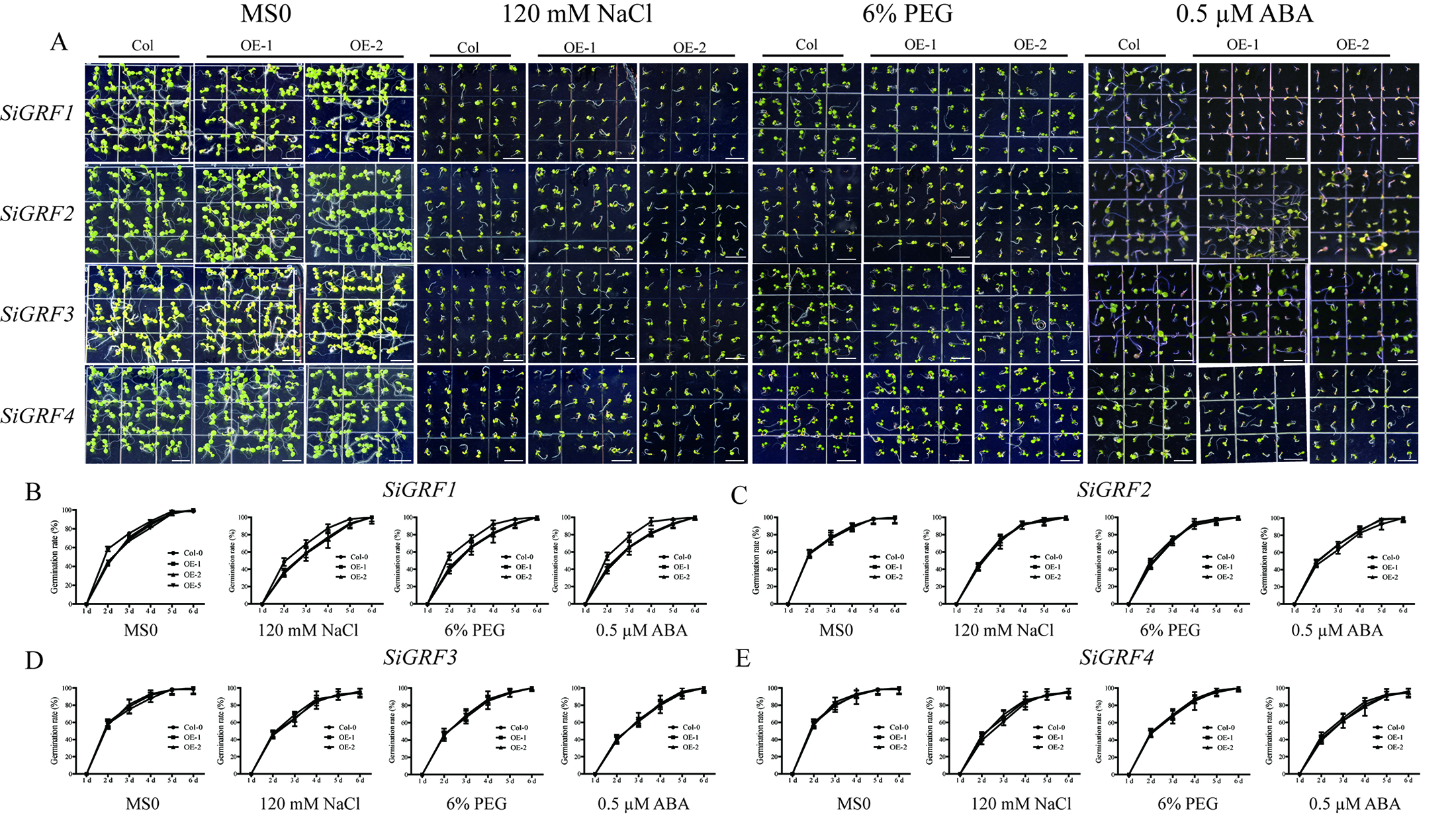

Supplement: FIGURE S1 — The seed germination rates of SiGRF1-OEs, SiGRF2-OEs, SiGRF3-OEs, and SiGRF4-OEs under the no-stress and stress treatments. (A–E) Germination rates of seeds after 6 days in the presence or absence of 120 mM NaCl, 6% (w/v) PEG 6000, and 0.5 μM ABA. At least 100 seeds of each line were counted for the measurement, and the seed number was recorded every 12 h post-incubation for visible radical emergence as a proxy for seed germination. Each treatment contained three independent replicates. [file Image_1.TIF]

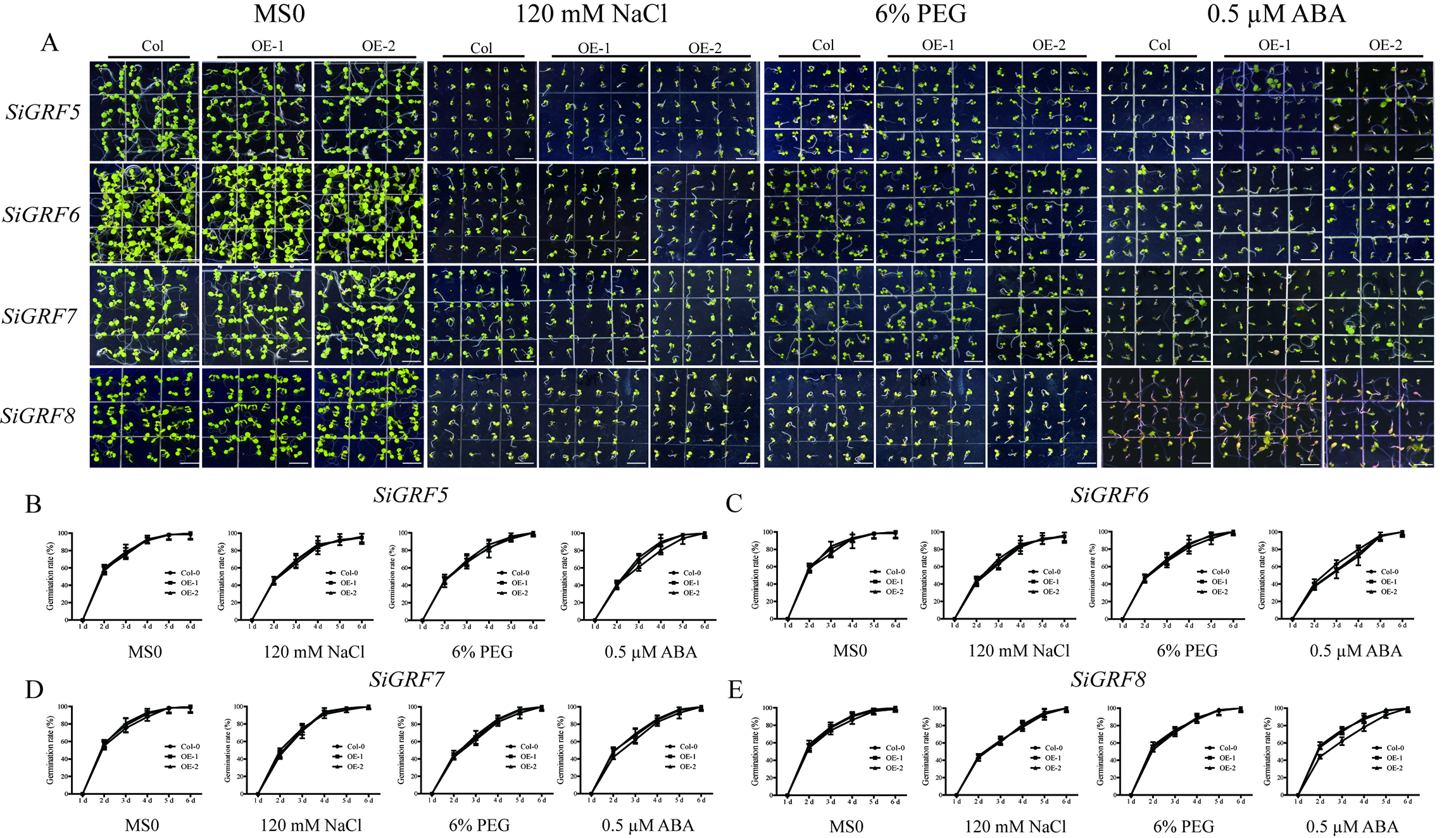

Supplement: FIGURE S2 — The seed germination rates of SiGRF5-OEs, SiGRF6-OEs, SiGRF7-OEs, and SiGRF8-OEs under the no-stress and stress treatments. (A–E) Germination rates of seeds after 6 days in the presence or absence of 120 mM NaCl, 6% (w/v) PEG 6000, and 0.5 μM ABA. At least 100 seeds of each line were counted for the measurement, and the seed number was recorded every 12 h post-incubation for visible radical emergence as a proxy for seed germination. Each treatment contained three independent replicates. [file Image_2.TIF]

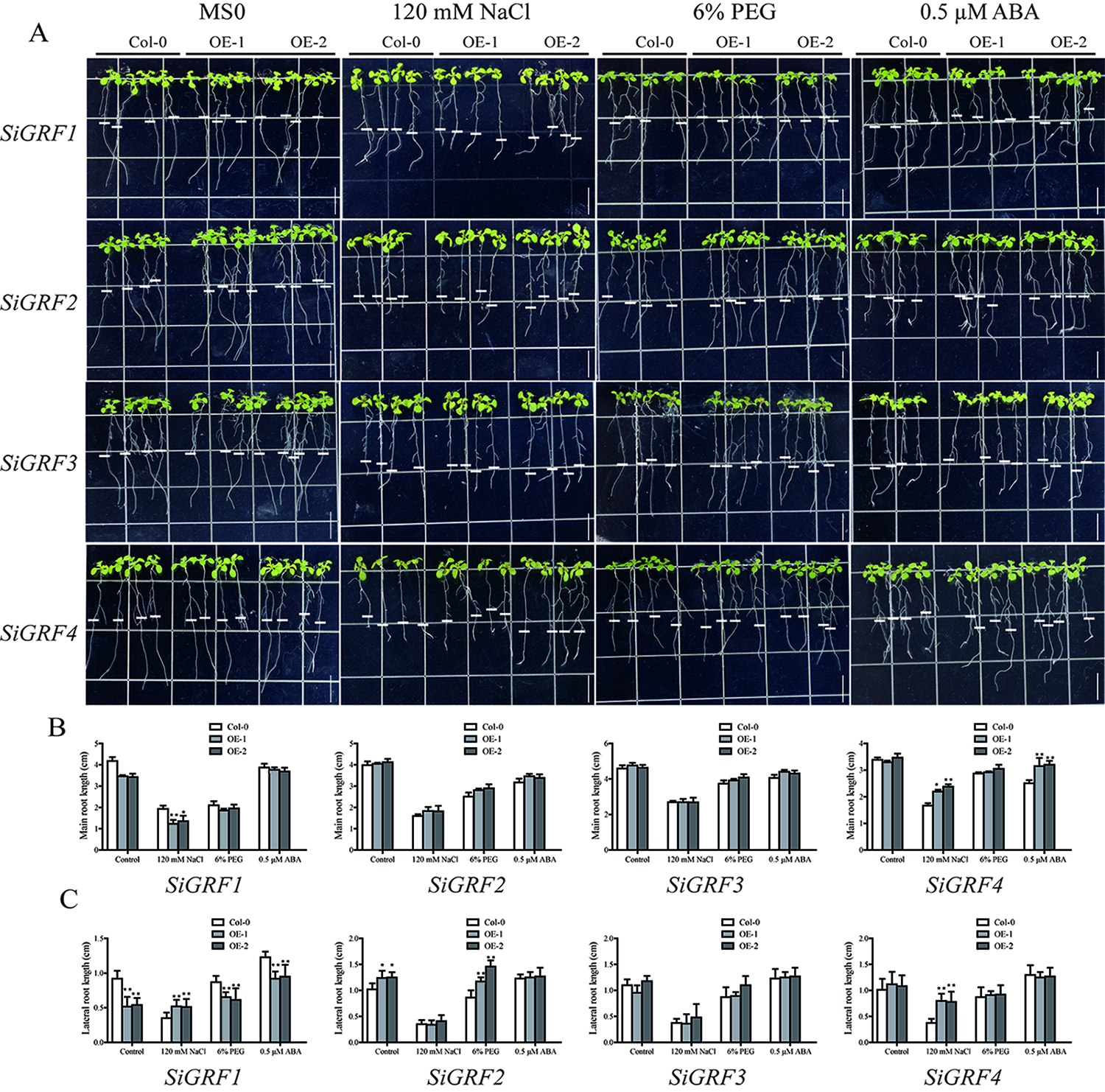

Supplement: FIGURE S3 — Phenotypic comparison of root lengths of SiGRF1-OE, SiGRF2-OE, SiGRF3-OE, and SiGRF4-OE plants grown on MS medium with or without treatment of 120 mM NaCl, 6% PEG, or 0.5 μM ABA. (A) Images were recorded on day 5 after the transfer of 5-day-old seedlings from 1/2 MS medium to plates containing 120 mM NaCl, 6% PEG, or 0.5 μM ABA, White solid line indicates that plants promote root growth. Bars = 1 cm. (B,C) Effect of different stress treatments on root growth in Col-0 and transgenic plants. Data represent means ± SD (n = 30). Students t-tests were used to generate the P-values. *P < 0.05; **P < 0.01. [file Image_3.TIF]

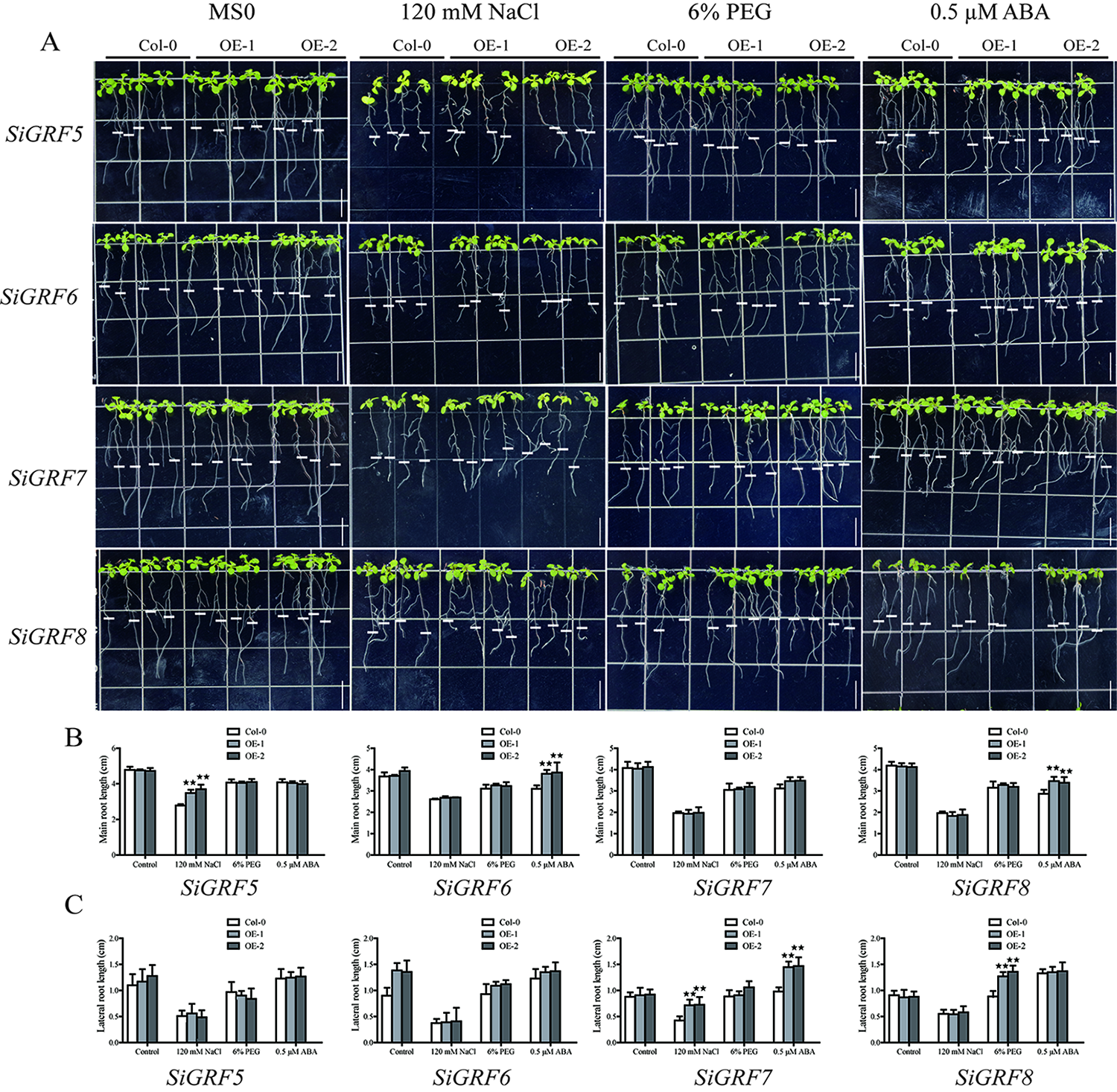

Supplement: FIGURE S4 — Phenotypic comparison of root lengths of SiGRF5-OEs, SiGRF6-OEs, SiGRF7-OEs, and SiGRF8-OEs plants grown on MS medium with or without treatment of 120 mM NaCl, 6% PEG, or 0.5 μM ABA. (A) Images were recorded on day 5 after the transfer of 5-day-old seedlings from 1/2 MS medium to plates containing 120 mM NaCl, 6% PEG, or 0.5 μM ABA, White solid line indicates that plants promote root growth. Bars = 1 cm. (B,C) Effect of different stress treatments on root growth in Col-0 and transgenic plants. Data represent means ± SD (n = 30). Students t-tests were used to generate the P-values. *P < 0.05; **P < 0.01. [file Image_4.TIF]
