# Supplementary material for: Over-Expression of a 14-3-3 Protein From Foxtail Millet Improves Plant Tolerance to Salinity Stress in Arabidopsis thaliana
Source: Front Plant Sci. 2020 Apr 15;11:449. doi: 10.3389/fpls.2020.00449 (PMC7174642; doi:10.3389/fpls.2020.00449)
Supplement: TABLE S3 — The sequences of primers used in the study. The sequences shown in lower case were added to generate a restriction enzyme site. [file Table_3.DOCX]

**Supplemental Table S3**. Primer sequences used in the study. The sequences shown in lower case were added to generate a restriction enzyme site.

| **Gene** | **Sequences** | **AGI number** |
| --- | --- | --- |
| **For gene cloning** | |  |
| SiGRF1F | GGGGATCC ATGTCGCAGCCTGCTGAGCTTT | Si020196m.g |
| SiGRF1R | ATGGATCC CTGCCCATCTCCAGACTCGCC |  |
| proSiGRF1F | ACGAATTC CCCTACTTTATGACAGATGTGG |  |
| proSiGRF1R | TCGAATTC CTTCACTGTTTATGGTTTCTAACAG |  |
| SiGRF2F | GGGGATCC ATGTCGAGGGAGGAGAATGTTTA | Si002635m.g |
| SiGRF2R | ATGGATCC CTGTCCCTCGCCAGCATCACCT |  |
| SiGRF3F | GGGGATCC ATGTCGAGGGAAGAGAATGTTT | Si014314m.g |
| SiGRF3R | ATGGATCC CTGTCCCTCGCCAGCATCGCCT |  |
| SiGRF4F | GGGGATCC ATGGCGGCGGCAGGAGGAGG | Si014301m.g |
| SiGRF4R | ATGGATCC CTCATCCTCGGGCTTGCTTG |  |
| SiGRF5F | GGGGATCC ATGTCGCGGGAGGAGAATGTC | Si014320m.g |
| SiGRF5R | ATGGATCC AGCCTCACCAGCATCGCCCTTG |  |
| SiGRF6F | GGGGATCC ATGGCATCAGCGGAGCTTTCC | Si010865m.g |
| SiGRF6R | ATGGATCC CTGCCCCTCGCTCGAGTCGTGC |  |
| SiGRF7F | GGGGATCC ATGTCGCCGTCGGAGCCGAC | Si026805m.g |
| SiGRF7R | ATGGATCC GGTTGTCACGCAGAGCTGCATG |  |
| SiGRF8F | GGGGATCC ATGGAGGAGCGGGAGAAGGTC | Si026782m.g |
| SiGRF8R | ATGGATCC ACCCTCCATGTCGATGTCATTAT |  |
| **For quantitative RT-PCR** | |  |
| RT-SiGRF1F | TTGACCTTTGGTGGACTGT | Si020196m.g |
| RT-SiGRF1R | ACGACCCTCTTCTTTCTGC |  |
| RT-SiGRF2F | AGGTATGAGGAGATGGTTGAGT | Si002635m.g |
| RT-SiGRF2R | TCGGCAGCAGTAGAAGAAG |  |
| RT-SiGRF3F | GCCAAGACCGTAGATGTTG | Si014314m.g |
| RT-SiGRF3R | CCTTTGACTCAGCAGCACT |  |
| RT-SiGRF4F | AAGATGAAGGGCGACTACC | Si014301m.g |
| RT-SiGRF4R | CAAGAGTATCCAGTTCCGAGA |  |
| RT-SiGRF5F | GAGCGTAACCTCCTGTCTGT | Si014320m.g |
| RT-SiGRF5R | AGCCTTATGGGATGGGTAG |  |
| RT-SiGRF6F | GCAATGAGGACCGTGTAAC | Si010865m.g |
| RT-SiGRF6R | AGTGCCAGACCAAGCCTAA |  |
| RT-SiGRF7F | CGAGTCCAAGGTCTTCTACC | Si026805m.g |
| RT-SiGRF7R | GGTGTCAGATGTCCATAGGG |  |
| RT-SiGRF8F | CTGGTGTGATGATAGATGCC | Si026782m.g |
| RT-SiGRF8R | AAGCCTGATTTGGTGAGC |  |
| SiActinF | AGAGCCACCACGACAAGTTC | Si036655m |
| SiActinR | CTGACGCCGAGGATATCCA |  |
| ACT2F | GGTAACATTGTGCTCAGTGGTGG | At3g18780 |
| ACT2R | AACGACCTTAATCTTCATGCTGC |  |
| RT-WRKY71F | TCGTTTCAAGATCCGTCGAT | AT1G29860 |
| RT-WRKY71F | TAGATGTTCAGCTGCCACGG |  |
| RT-FTF | CTTGGCAGGCAAACAGTGTATGCAC | AT1G65480 |
| RT-FTR | GCCACTCTCCCTCTGACAATTGTAGA |  |
| RT-LFYF | ATCGCTTGTCGTCATGGCTG | AT5G61850 |
| RT-LFYR | GCAACCGCATTGTTCCGCTC |  |
| RT-FULF | GAGAAGAAAACGGGTCAGCAAG | AT5G60910 |
| RT-FULR | AAAGCCATCTCTGGAGGAGGTTA |  |
| RT-OLEO1F | TGGTTCCCTCCTTGTTCTC | AT4G25140 |
| RT-OLEO1R | GCACTGTCCAACTTGTCTGA |  |
| RT-OLEO2F | TTACAATGGCGGATACACAC | AT5G40420 |
| RT-OLEO2R | CAGGGACTCCAATCAACAG |  |
| RT-OLEO4F | TCAGCCAAACTACGAAGATG | AT3G27660 |
| RT-OLEO4R | CAAACCAGAAGCCAAGATTC |  |
| RT-AT3G01570F | TCCTTTATCCTCAGAGTGGC | AT3G01570 |
| RT-AT3G01570R | TCAGCCAAACGATGCTTC |  |
| RT-ELIP1F | GCAACAGCATCGTTCAAC | AT3G22840 |
| RT-ELIP1R | AGTCTTCATTCGTGGGTCC |  |
| RT-PXMT1F | AGAGCATTGTTGGAAGCG | AT1G66700 |
| RT-PXMT1R | GGAAGTGTCTGGAAGAGAGTG |  |
| RT-EPR1F | GTATGAGACTGATAGTGGTCACG | AT2G27380 |
| RT-EPR1R | GACTGTAAGTTGGTGTCGGG |  |
| RT-CRU3F | AACCAACAAGACAGCAGAGG | AT4G28520 |
| RT-CRU3R | TGACATACTCCAAGATGGGC |  |
| RT-AGL67F | AAGCCACCAACTCCAATGTC | AT1G77950 |
| RT-AGL67R | TTCGCAGGACTTTCTCAACA |  |
| RT-AT3G56350F | TTCGTTGCCATCTTCGCTAC | AT3G56350 |
| RT-AT3G56350R | AGGCTGTTGAGGGCTTTGTT |  |
| RT-MAF4F | TTCTTTGTGAATCCTCCGTC | AT5G65070 |
| RT-MAF4R | TAGCAACTCCTTGTGCGAAA |  |
| RT-AT4G22460F | TTCACTCTCACTACTGCCACTC | AT4G22460 |
| RT-AT4G22460R | AGGGTAAGGTTGATGCCAAG |  |
| RT-AT3G21230F | TCTTGCCGTTGCTTACCTTG | AT3G21230 |
| RT-AT3G21230R | AATCATCACTCCCTTTGGAAGTC |  |
| RT-ADPG1F | ACAATCCCAAACAAGACAGG | AT3G57510 |
| RT-ADPG1R | CACTAACGGTTGATGCTTCC |  |
